# State-of-the-art and novel developments of in vivo haploid technologies

**DOI:** 10.1007/s00122-018-3261-9

**Published:** 2018-12-19

**Authors:** Kamila Kalinowska, Sindy Chamas, Katharina Unkel, Dmitri Demidov, Inna Lermontova, Thomas Dresselhaus, Jochen Kumlehn, Frank Dunemann, Andreas Houben

**Affiliations:** 1 Biochemie-Zentrum Regensburg, University of Regensburg, Universitätsstraße 31, 93053 Regensburg, Germany; 2 Leibniz Institute of Plant Genetics and Crop Plant Research (IPK) Gatersleben, Corrensstraße 3, 06466 Stadt Seeland, Germany; 3 Institute for Breeding Research on Horticultural Crops, Federal Research Centre for Cultivated Plants, Julius Kühn-Institute (JKI), Erwin-Baur-Str. 27, 06484 Quedlinburg, Germany

## Abstract

The ability to generate (doubled) haploid plants significantly accelerates the crop breeding process. Haploids have been induced mainly through the generation of plants from cultivated gametophic (haploid) cells and tissues, i.e., in vitro haploid technologies, or through the selective loss of a parental chromosome set upon inter- or intraspecific hybridization. Here, we focus our review on the mechanisms responsible for the in vivo formation of haploids in the context of inter- and intraspecific hybridization. The application of a modified CENH3 for uniparental genome elimination, the IG1 system used for paternal as well as the BBM-like and the patatin-like phospholipase essential for maternal haploidy induction are discussed in detail.

## Introduction

The ability to generate haploids and subsequently induce whole genome duplication has provided a strategy to significantly accelerate the crop breeding process. The major advantage of the resulting doubled haploids to breeders lies in the simultaneous genetic fixation at every locus within a single generational step. This avoids the time-consuming conventional requirement for extensive selfing or backcrossing before breeding inbred lines can be obtained. Once a doubled haploid line has been created, its genotype can be identically reproduced and rapidly multiplied, allowing breeders to evaluate as many traits as they need to handle using genetically homogeneous material and an extraordinary selection efficacy already at the very early stage of the breeding cycle. The result is a substantial saving in both time and resources as compared to conventional practice. However, in many crop species, viable haploid technology is not yet available or only applicable to a limited number of genotypes and at exceedingly high costs, although the generation of haploid plants can be achieved by various approaches [reviewed by Dwivedi et al. (2015), Ishii et al. (2016), Kumlehn (2014) and Shen et al. (2015)]. Due to the application limits of current haploid technology, plant breeders are highly interested in any methodological improvements as well as in novel principles of haploidization.

**Fig. 1:**
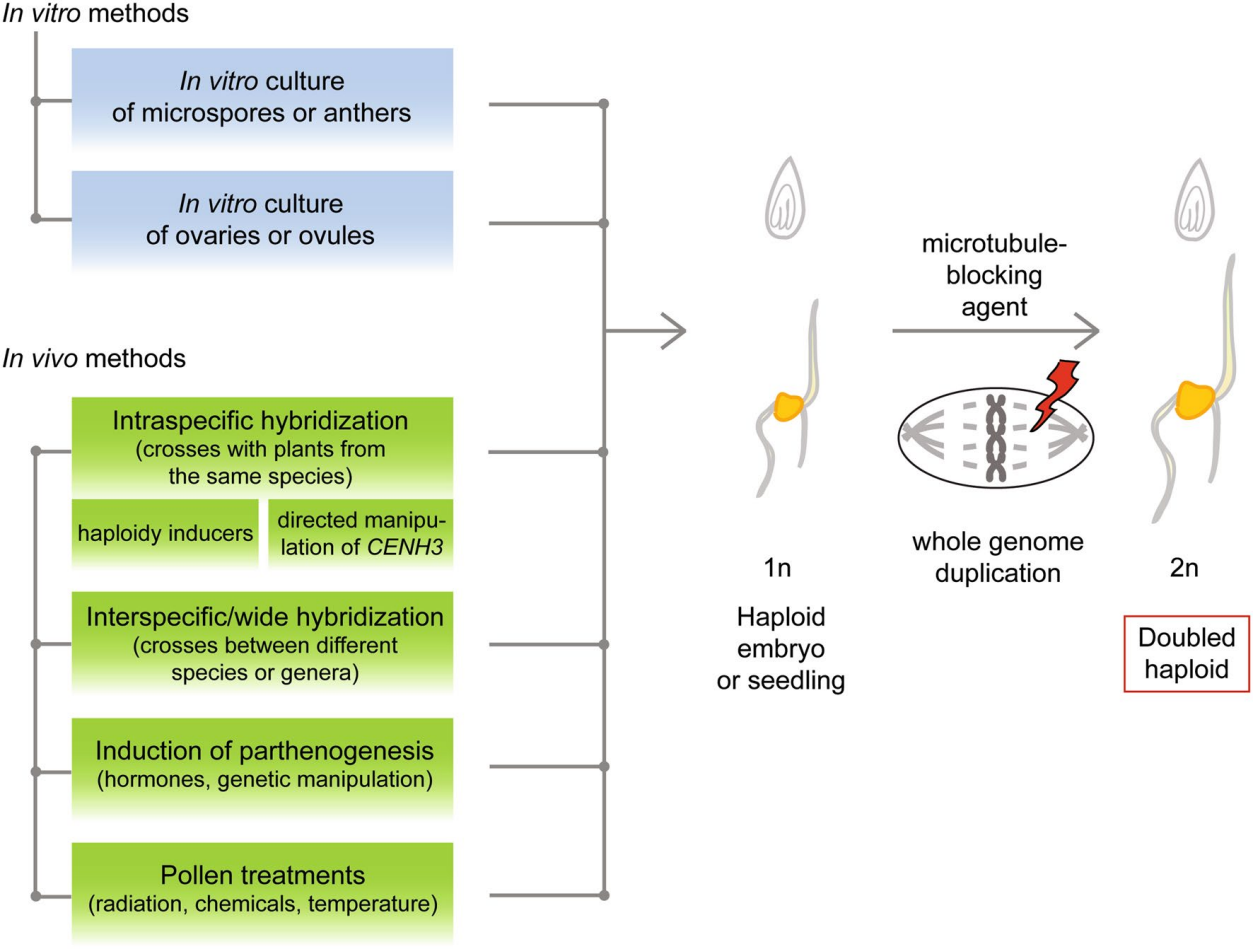
Overview of methods for haploid induction in plants. In vitro methods for haploid induction involve the cultivation of male or female gametophytic cells derived from immature anthers (paternal haploids) and ovaries/ovules (maternal haploids), respectively. In vivo induction of maternal haploids can be initiated by pollination with pollen of the same species (intraspecific hybridization), where classical haploidy inducers or plants carrying mutations within *CENH3* are typically used. Pollination with pollen of a wild relative or unrelated species is named as interspecific or wide hybridization. Parthenogenesis was also reported in plants treated with hormones or after genetic manipulations. Haploidy may also occur after pollination with (defective) pollen treated by radiation, chemicals or at high temperature. In some in situ methods where the development of endosperm does not take place, embryo rescue is required. Haploid embryos or seedlings undergo spontaneous genome duplication or are treated with a microtubule-blocking agent such as colchicine to induce the reduplication of the haploid genome and the generation of doubled haploid plants

In the most conventional format of haploid technology, plants originate directly from gametophytic (haploid) cells contained in a variety of explants cultivated in vitro, e.g., pistils, ovules, anthers or isolated immature pollen (typically microspores, more rarely early bicellular pollen). In dependence on whether the regenerated plants inherit their haploid genome from female or male gametophytes (embryo sac and pollen), they are termed as maternal or paternal haploids, respectively. Alternatively, haploids can be produced via intra- or interspecific crosses entailing uniparental genome elimination that typically takes place during early zygotic embryogenesis. As a further opportunity, parthenogenesis was reported in plants treated with hormones or after genetic manipulations. Haploidy may also occur after pollination with pollen treated by radiation, chemicals or at high temperature (Fig. 1).

The present review focuses on the mechanisms underlying the selective elimination of one of the genomes during the early cell divisions of an embryo and describes in some detail methods for haploid induction that involve targeted centromere manipulation. In addition, progress is discussed on the recent identification of genes contributing to genome stability and whose modification thus can also entail the induction of haploidy.

## Generation of haploids via intraspecific hybridization

### IG1-system for paternal haploid induction in maize

Haploid induction technology is used in maize breeding since decades. Two approaches are generally used for in vivo haploid induction: the first method involves the *indeterminate gametophyte1* (*ig1*) mutant and the second approach depends on Stock6-derived lines. The *ig1* mutation was first observed as a spontaneous mutation in the inbred line Wisconsin-23 (W23) leading to about 3% haploid induction rates (HIR) of paternal haploids. The inducer containing *ig1* is used as female parent. Paternal haploids thus contain the cytoplasm of the female inducer and the haploid genome of the pollen donor. Using *ig1* mutants, maternal haploids were also obtained (Kermicle 1969), albeit the frequency (ca. 0.1%) was dependent on the genetic background and too low to be clearly distinguishable from the spontaneous formation of haploids derived from crossings of wild-type plants (Chase 1949). The ability of producing paternal haploids has been utilized in maize breeding to transfer chromosomes from one variety of maize to the cytoplasm of another variety (Ren et al. 2017).

Fine mapping studies revealed that *ig1* is localized on maize chromosome 3 and encodes a LATERAL ORGAN BOUNDARIES (LOB)-domain protein (Evans 2007). LOB domain-containing proteins belong to a large plant-specific family of transcription factors that were shown to be essential for lateral organ development in higher plants (Husbands et al. 2007). The closest Arabidopsis homolog of IG1, ASYMMETRIC LEAVES 2 (AS2), is important for a correct leaf left–right symmetry and proper formation of leaf primary and secondary veins (Semiarti et al. 2001). In maize, *ig1* mutant plants of inbred line W23 contain a Hopscotch retrotransposon insertion within the second exon of the gene, upstream of the encoded LOB domain (Evans 2007). Though *ig1* mutants are viable, a number of phenotypes were observed occurring during female gametophyte (embryo sac) development. Uncellularized *ig1* embryo sacs contain an increased number of nuclei due to mitosis synchronization failures and abnormal microtubular behavior. After embryo sac cellularization, individual cells in its micropylar region that normally contains one egg and two synergid cells continue to undergo mitosis. Irregular positioning of nuclei, asynchronous microtubular patterns in different pairs of nuclei, and abnormal phragmoplasts have also been observed (Huang and Sheridan 1996). The proliferative phase is prolonged, leading to formation of extra egg cells, extra central cells, and extra polar nuclei within central cells. Three or more synergid cells are present in about 75% of embryo sacs that can be penetrated by two or more pollen tubes. Multiple fusions between sperm and egg cells have been described to occur in the same embryo sac. This can entail the generation of multiple embryos. The above-mentioned phenotypes suggest that wild-type *IG1* functions to promote the switch from proliferation to differentiation in the maize embryo sac (Evans 2007; Guo et al. 2004; Huang and Sheridan 1996). This hypothesis is supported by the finding that *IG1* is a target of egg cell secreted differentiation factor EAL1 signaling pathway (Krohn et al. 2012). Due to their abnormal structure, many defective *ig1* embryo sacs give rise to abnormal kernels containing two or more embryos, miniature endosperms, or cause early seed abortion (Evans 2007; Kermicle 1969).

The exact mechanisms through which *ig1* mutations lead to haploid induction are not understood. LOB domain-containing proteins are known to interact with members of the basic helix-loop-helix (bHLH) family of transcription factors and regulate the expression of a number of downstream genes. The Arabidopsis homolog AS2 has been shown to repress the expression of the *knotted1*-*like* homeobox (*knox*) genes *AtKNAT1*, *AtKNAT2*, and *AtKNAT6* in leaf primordia (Semiarti et al. 2001). Downregulation of the rice homolog *OsIG1* was shown to affect expression of genes such as *OsEG1*, *OsMADS6* and *OsMADS1* in the embryo sac. This indicates that *Os*IG1 might participate in regulating floral organ and female gametophyte development-associated genes (Zhang et al. 2015). Downregulation of *KNOX8* was also reported for maize *ig1* embryo sacs indicating the existence of conserved gene regulatory mechanisms (Evans 2007). However, the exact mechanisms remain unclear which renders the transfer the IG1-system to other crops difficult.

### Stock6-derived maternal haploid induction systems of maize

The second above-mentioned approach that is broadly utilized for in vivo haploidy induction in maize involves Stock6-derived haploidy inducer lines. Stock6 is an inbred line originally reported to cause haploid induction rates of 2.3–3.2% (Coe 1959). The haploid-inducing capacity of this founder line was subsequently introduced by different research groups and breeders into various genetic backgrounds. Further hybrids were established including a cross between Stock6 and the above-mentioned *ig* mutant in W23, which became a basis for many currently used inducer lines such as RWS, UH400, MHI and PHI showing a haploid induction rate of around 7–16% (Hu et al. 2016; Prigge et al. 2012).

Progeny from a cross between the inducer line PK6 (haploid induction rate 6%) and the non-inducer line DH99 was used to take a mapping approach, which resulted in the identification of a QTL associated with haploid induction at chromosome 1 bin 1.04. It was named as *gynogenesis inducer 1* (*ggi1*). Incomplete penetration and segregation distortion were observed probably due to a lower rate of transmission via male gametes (Barret et al. 2008). A more recent study, however, revealed that haploid induction ability of Stock6-derived lines is a complex quantitative trait controlled by several loci. Four biparental populations were analyzed, where an inbred UH400 was used as pollinator against another haploid-inducing line, namely CAUHOI, and three non-inducer lines, the temperate line 1680 and two tropical lines CML395 and CML495. This resulted in the identification of eight QTL on six chromosomes. In agreement with previous studies, a mandatory QTL for haploid induction was detected on chromosome 1 to bin 1.04, which was named *qtl for hir 1* (*qhir1*) (Prigge et al. 2012). Another major QTL is localized on chromosome 9 (*qhir8*). This explains the observed 20% genotypic variance and strengthens the idea that *qhir1* is modulated by further loci which are not capable to cause haploid induction on their own (Liu et al. 2015; Prigge et al. 2012).

By adapting the progeny test method for fine mapping, the 50.3 Mbp-large *qhir1* locus was further narrowed down to 243 kbp. In these experiments, inducer line UH400 was used as pollinator of line 1680. The progeny was selfed twice and then pollinated to hybrid ZD958, which resulted in the identification of haploids using the *R1*-*nj* marker derived from UH400. The new QTL was called *qhir1* and was shown to be essential for haploid induction (Dong et al. 2013). In another effort to identify a sub-region responsible for haploid induction within *qhir1*, a genome-wide association study (GWAS) was conducted using 53 haploidy induction lines and 1482 non-inducers. A QTL of 3.97 Mbp common for all inducers, which was absent in all non-inducers and which does not overlap with *qhir11*, was identified and designated as *qhir12* (Hu et al. 2016). In order to dissect the sub-region of *qhir1*, another group used selfed progeny of recombinants of a large F2 population derived from crossing the tropical non-inducer CML269 with a tropical haploidy inducer line TAIL8. Out of two *qhir1* sub-regions, the region containing *qhir11* had a significant effect on the generation of maternal haploids independent of the *qhir12* allele, whereas the “inducer” *qhir12* allele did not cause haploid induction higher than the spontaneous rate observed in case of wild-type cobs. In addition, the region containing *qhir11* was reported to display segregation distortion and kernel abortion, which are characteristic for haploidy inducer lines (Nair et al. 2017).

More recently, three groups independently published that a patatin-like phospholipase located in *qhir11* is essential for haploid induction in Stock6-derived lines. The phospholipase was named MATRILINEAL (MTL), PLA1 (PHOSPHOLIPASE A1) and NOT LIKE DAD (NLD) (Gilles et al. 2017a; Kelliher et al. 2017; Liu et al. 2017). A 4-bp insertion within the fourth exon of the *MTL/PLA1/NLD* gene was detected in inducer lines for which a protein truncation at amino acid 380 is characteristic. As a result of the insertion, this site is followed by 20 altered amino acid residues and a premature stop codon that truncates the protein by 29 amino acids. *MTL/PLA1/NLD* mutants were shown to induce haploids in vivo, which is accompanied with the typical side effects such as kernel abortion and segregation distortion (Gilles et al. 2017a; Kelliher et al. 2017; Liu et al. 2017). For example, CRISPR-Cas9 lines targeting the first exon of the phospholipase showed an average of 2% haploid induction and 9–14% kernel abortion (Liu et al. 2017). Small deletions nearby the site of the 4-bp insertion in Stock6-derived lines induced via transcription-activator-like effector nuclease (TALEN)-mediated targeted mutagenesis led to haploid induction of 4–12% (average 6.65%) (Kelliher et al. 2017).

Gene expression studies revealed that *MTL/PLA1/NLD* activity in maize is confined to mature pollen grains and pollen tubes (Gilles et al. 2017a). Wild-type MTL/PLA1/NLD containing a C-terminally fused fluorophore localizes to the cytoplasm and plasma membrane (PM) of sperm cells in germinated pollen tubes. Notably, the truncated version is not expressed at detectable levels irrespective of whether an endogenous or constitutive promoter was chosen for its expression (Gilles et al. 2017a; Kelliher et al. 2017). This observation suggests instability of the mutated variant in maize cells.

MTL/PLA1/NLD belongs to the phospholipase A (PLA) superfamily of enzymes that catalyze the hydrolysis of acyl groups from phospholipids to produce free fatty acids and lysophospholipids. Plant PLAs are divided into three families, PLA1s, PLA2s and patatin-like phospholipases (pPLAs/PLPs), depending on the specificity of the hydrolysis site (Chen et al. 2013; Scherer et al. 2010). MTL/PLA1/NLD belongs to the plant pPLA/PLP family (Gilles et al. 2017a; Kelliher et al. 2017), which is further divided into three sub-classes based on sequence comparisons. Phospholipases of class I show the highest homology to animal iPLAs and contain a C-terminal leucine-rich repeat domain and an N-terminal domain of unknown function. Proteins from class II are more related to patatins than those of class III, in which the catalytic center is different and consists of a motif GxGxG as part of the catalytic dyad (Chen et al. 2013; Scherer et al. 2010). Phylogenetic analysis revealed that MTL/PLA1/NLD belongs to class II pPLAs/PLPs (Gilles et al. 2017a).

The mechanisms through which the C-terminal truncation of MTL/PLA1/NLD leads to haploidy induction are not yet understood. It is also still not entirely clear how the truncation affects the function of MTL/PLA1/NLD. It was suggested that the lost capability of membrane binding may play an essential role (Gilles et al. 2017a). When expressed in Arabidopsis root epidermis under the constitutive *AtUBQ10* promoter, wild-type MTL/PLA1/NLD-CITRINE localized to cytosol and plasma membrane (PM), with signals assigned to small intracellular compartments. The truncated version lost its PM localization and signals were observed exclusively in the cytosol. Further support is provided by the fact that potential S-palmitoylation or S-farnesylation sites at C423 are lost in the truncated protein (Gilles et al. 2017a).

Another possible consequence of the C-terminal protein truncation is that a potential phosphorylation site is removed, which might have a strong effect on the protein’s ability to be activated during cellular signaling. Activity of two of the closest Arabidopsis homologs of MTL/PLA1/NLD, *At*PLAIVA/PLP1 and *At*PLAIVB/PLP5, was shown to be affected by phosphorylation at the C-terminus by calcium-dependent protein kinases (CDPKs). Deletion of the last 16 amino acids containing 4 serines at the C-terminus of *At*PLAIVA/PLP1 and *At*PLAIVB/PLP5 completely abolished their phosphorylation by *At*CDPK3. A mutation of Ser399, which is conserved in MTL/PLA1/NLD (corresponding to Ser402), decreased the efficiency of protein phosphorylation dramatically. Phosphorylation of *At*PLAIVA/PLP1 and *At*PLAIVB/PLP5 was further shown to significantly affect their activity and specificity toward various substrates (Rietz et al. 2010). It is thus well possible that MTL/PLA1/NLD also requires phosphorylation at its C-terminus by a yet unknown sperm cell-specific kinase for proper signal transduction in cell signaling responses during fertilization.

Anyhow, the modulation of MTL/PLA1/NLD function, independent of whether its localization, activity or substrate specificity are affected, lead to haploid induction and kernel abortion. It was suggested that these phenomena are (i) either associated with the failure of fertilization while zygote identity is activated in the egg cell (resulting in haploid induction via parthenogenesis) and/or endosperm identity in the central cell (resulting in defective endosperm followed by kernel abortion), or (ii) by post-zygotic uniparental elimination of the paternal genome. In conclusion, it is still unknown how haploid induction is mechanistically triggered upon pollination by Stock6-derived lines and *mtl/pla1/nld* mutants. Investigations to study both uniparental chromosome elimination (Li et al. 2009, 2017; Zhang et al. 2008; Zhao et al. 2013) and single fertilization (Tian et al. 2018) are underway.

Another interesting question is how the other main QTL found in maize haploidy inducers, *qhir8* (Liu et al. 2015; Prigge et al. 2012), affects the function of MTL/PLA1/NLD. *qhir8* was fine-mapped through the analysis of progeny derived from the cross of two inducers containing a fixed *qhir1* region, CAUHOI (2% haploid induction) and UH400 (8% haploid induction). It was found that haploid induction of F2 plants homozygous for UH400 at *qhir8* was significantly higher than that of F2 plants homozygous for CAUHOI at *qhir8*. The mean induction rate of heterozygous F2 plants was reported to be in-between the two homozygous classes. These results confirmed that *qhir8* has the potential to double the haploid induction rate caused by *qhir1* (Liu et al. 2015). Future studies will elucidate the detailed molecular mechanisms of MTL/PLA1/NLD activity in wild-type and mutant plants. It will also be exciting to find out whether site-directed modification of orthologous genes in other crops may result in maternal haploid induction allowing the transfer of the approach to lacking viable haploid induction systems. A first step has been made recently to engineer haploid induction in rice: a pollen-specific *MTL/PLA1/NLD* homolog (*OspPLAIIφ* or *OsMATL*) was subjected to site-directed modification using RNA-guided Cas endonuclease technology. Knockout mutants showed reduced seed set and a haploid induction of up to 6% (Yao et al. 2018). This first report is very promising, and it can be expected that haploid induction will soon also be possible in other grasses. Major future steps are needed to increase haploid induction and to transfer this important breeding trait also to eudicotyledonous crops.

Although the use of efficient inducer lines is essential to obtain sufficient haploidy induction rates, the importance of the “donor” genotype should not be ignored. Already in 1949 Chase observed that genotypes of both parent plants have a strong influence on spontaneous haploidy induction rates in maize (Chase 1949). Additionally, in some cases the dominance of factors conferring low frequency of haploidy has been reported (Coe 1959; Lashermes and Beckert 1988).

For Stock 6-derived lines, Eder and Chalyk (2002) tested the influence of source germplasm on haploidy induction rates of two paternal inducer lines, MHI and M741H. Among the tested maternal genotypes were four European flint lines, eleven dent lines and five flint × dent hybrids. Maternal haploids could be induced for all genotypes, though at variable rates with none of the genetic pools showing a significant advantage over others. Also for tropical maize, the source germplasm has been shown to be of great importance leading together with environmental conditions to differences of HIR between 2.9 and 9.7% for the same paternal inducer (Kebede et al. 2011). Efforts to map the regions determining the maternal contribution to haploidy induction suggest existence of two loci on chromosomes 1 and 3, termed *qtl for maternal hir 1* (*qmhir1*) and *qmhir2*, respectively, and explain up to 20% of the phenotypic variation (Wu et al. 2014). Also for *indeterminate gametophyte 1*, a certain correlation between the genotype of the pollinator and the frequency of maternal and paternal haploids was observed, though not as straightforward as for Stock6-derived inducers (Lashermes and Beckert 1988).

Finally, other aspects such as the generation of the haploidy inducer and the “donor” (Li et al. 2008), field or greenhouse conditions (Eder and Chalyk 2002; Roeber et al. 2005) or season (Kebede et al. 2011) have also been shown to play significant roles during haploidy induction and should be taken into consideration during planning of crossing events both in research and breeding. The importance of the “donor” genotype and of plant growth and pollination conditions adds another layer to the complicated aspect of in vivo haploidy induction. It is the limitation in the use of haploidy inducers, and can be applied to be of advantage.

## Generation of haploids via induction of parthenogenesis—BBM-triggered maternal haploid induction

As discussed above, it is still unclear whether the MTL/PLA1/NLD-system leads to uniparental chromosome elimination and/or induction of parthenogenesis. Together with apomeiosis leading to unreduced gametes and autonomous endosperm development in some species, parthenogenesis (development of an embryo from an unfertilized egg cell) is also a key component of apomixis (Ronceret and Vielle-Calzada 2015). Apomixis (asexual reproduction through seeds) has evolved independently in numerous plant species, but genes associated with the components of apomixis remained elusive. Recently, a major gene responsible for the parthenogenesis trait was discovered in the apomictic grass *Pennisetum squamulatum* (pearl millet). The gene named as *PsASGR*-*BABY BOOM*-*like* (*PsASGR*-*BBML*) is expressed in egg cells before fertilization and could be used to successfully induce parthenogenesis at a rate of about 35% in sexual pearl millet (Conner et al. 2015). In species like maize and rice, homologous genes are absent in egg cells, but are quickly activated de novo in zygotes after fertilization (Anderson et al. 2017; Chen et al. 2017). For this reason, it is not surprising that the *PsASGR*-*BBML* gene could also be used to induce parthenogenesis in maize and rice leading to the formation of haploid embryos at a rate of 25–89% (Conner et al. 2017). Both, the endogenous pearl millet *PsASGR*-*BBML* promoter as well as an egg cell-specific promoter from Arabidopsis was used to generate haploid embryos. Notably, *PsASGR*-*BBML* was shown to be insufficient to induce parthenogenesis in the model plant *Arabidopsis thaliana.* A similar observation was made vice versa: BABY BOOM (BBM), a member of the AP2/ERF family of transcription factors, was first discovered in *Brassica napus* (oilseed rape) and shown to be capable of inducing somatic embryos on seedlings in Brassica and Arabidopsis after ectopic overexpression (Boutilier et al. 2002). It was suggested that BBM represents an embryonic cell proliferation/morphogenesis factor. First attempts to transfer *BBM* from Brassica to maize using an embryo sac-specific promoter derived from *ES1*-*4* genes (Cordts et al. 2001) failed as they did not result in induction of parthenogenetic embryo development (S. Amien and T. Dresselhaus, unpublished results). Genetic programs during early embryogenesis are different in eudicots and monocots (Zhao et al. 2017). Moreover, the various members of the BBML family of transcription factor genes likely possess different targets and may thus not be interchangeable. Therefore, it is intriguing to find out whether zygotic BBML genes from maize can induce parthenogenesis once expressed from an egg and/or embryo sac-specific promoter, and whether zygotic BBML genes exist in eudicots and can be used for haploid induction in Brassica and other eudicotyledonous crops.

## Generation of haploids via interspecific hybridization

Upon interspecific fertilization, two different parental genomes are combined within one nucleus, which in most cases is embedded within the maternal cytoplasm. Such a novel genomic constitution may result in intergenomic parental conflicts and as a consequence a genomic and epigenetic reorganization of the genomes occurs (Riddle and Birchler 2003). Even if in most cases the parental genomes remain combined at least partly after a successful fertilization, uniparental genome elimination has been found in more than 100 different species combinations, including 75 and 26 examples for mono- and eudicot species, respectively (Ishii et al. 2016). For example, haploid wheat can be obtained after pollination of wheat with pollen of either *H. vulgare, Z. mays*, *Coix lachrymajobi*, *Teosinte, Trypsacum dactyloides, Pennisetum glaucum, Imperata cylindrical* or *Sorghum bicolor* (Ishii et al. 2016). Most monocot species hybrids required in vitro tissue culture to rescue developing embryos due to endosperm abortion. An extremely high frequency of doubled haploids was recently reported for *Brassica napus*. It was shown that pollination of allohexaploid *B. napus* (AAAACC) with pollen of artificially generated allooctaploid *Brassica* (AAAACCCC) resulted in up to 98% doubled haploids (Fu et al. 2018).

Depending on the species combination, the process of uniparental chromosome elimination can be as fast as in the context of the first zygotic division or as slow as three weeks after fertilization as was demonstrated in the combinations wheat × *Imperata cylindrica* (Mukai et al. 2015) and wheat × *Pennisetum glaucum* (Gernand et al. 2005), respectively. The process of haploidization via wide hybridization itself is best studied for the combination *Hordeum vulgare* × *H. bulbosum.* Davies (1956) obtained barley-like diploid plants from a cross between tetraploid *H. bulbosum* (female) and tetraploid *H. vulgare* (male) and suggested they originated by male parthenogenesis. Later reports of Symko (1969) and Lange (1969;1971) independently presented the hypothesis of chromosome elimination as a mechanism of haploid barley production. This was confirmed by Kasha and Kao (1970), because hybridizations of diploid *H. vulgare* with diploid *H. bulbosum* resulted in the production of haploid *H. vulgare* plants through the complete loss of the *H. bulbosum* genome. The application of the so-called ‘*bulbosum’* method was promoted by the elaboration of robust protocols including embryo rescue techniques able to generate haploid embryos in up to 30% of florets pollinated (reviewed in (Kumlehn 2014)).

Haploid formation due to uniparental chromosome elimination in barley is known to depend on genetic factors (Ho and Kasha 1975) and temperature after fertilization (Pickering 1985). A temperature above 18 °C during the early embryo development can promote chromosome elimination. In addition, Kasha and Kao (1970) suggested that the genome balance between the parental species is important in chromosome stability. Chromosomes of *H. bulbosum* are eliminated over several days after pollination (Bennett and Rees 1967; Sanei et al. 2011) independent of the crossing direction, while hybrids combining both sets of parental chromosomes can be obtained (Humphreys 1978). A tissue-specific alternative elimination of whole parental genomes was observed in the embryo and endosperm of *H. marinum* × *H. vulgare* crosses (Finch 1983).

Cytological studies revealed that the uniparental chromosome loss occurs preferentially by formation of micronuclei at mitosis during early hybrid embryo development (Kasha and Kao 1970). Chromosomes destined for elimination did often not congregate properly at metaphase and were lagging behind other chromosomes at anaphase (Laurie and Bennett 1989). These observations are consistent with the classical mechanism of micronucleus formation, which involves the enclosure of lagging chromosome fragments during reformation of nuclear membranes at the end of mitosis. More recently, detection of the centromeric histone H3 variant CENH3 in *H. vulgare* × *H. bulbosum* embryos demonstrated that uniparental centromere inactivation is involved in the process of uniparental chromosome elimination (Sanei et al. 2011). Active centromeres of *H. vulgare* and of *H. bulbosum* were CENH3-positive, while inactive *H. bulbosum* centromeres were CENH3-negative or the amount of CENH3 was reduced in unstable hybrids. Likely sperm-derived centromere-incorporated CENH3 proteins provide residual kinetochore function of *H. bulbosum* until it falls below a level critical for correct chromosome segregation, which eventually results in chromosome elimination. If pre-existing CENH3 is partitioned equally between duplicated sister centromeres and no de novo incorporation of CENH3 into *H. bulbosum* centromeres occurs, its amount will be approximately halved with each cell division. In humans, even 10% of the endogenous CENH3 can aid efficient kinetochore assembly (Liu et al. 2006). If a zygotic resetting of CENH3 also exists in grasses as demonstrated in *A. thaliana* (Ingouff et al. 2010), an active removal of both parental CENH3s prior to the first zygote division would occur and the reactivation of *H. bulbosum* centromeres in unstable hybrids would be diminished.

Uniparental inactivation of *CENH3* is not the cause of centromere inactivation in unstable barley × *H. bulbosum* hybrids (Sanei et al. 2011). It is assumed that in unstable hybrids, the incorporation of CENH3 into the centromeres of the paternal genome is impaired. The regulation of CENH3 loading and assembly into centromeres is mediated by a number of proteins and the erroneous function of any of these may result in a non-functional centromere (reviewed in (Gohard et al. 2014)). However, except KNL2 (Lermontova et al. 2013) and GIPs (Batzenschlager et al. 2015) of *A. thaliana*, no other protein involved in the establishment and maintenance of CENH3-specific chromatin has thus far been identified in plants.

The centromere-dependent process of elimination in Triticeae (like *T. aestivum, T. spelta, T. durum*, Triticale, *T. monococcum*, S*ecale cereale* and *Avena sativa*) × pearl millet combinations is likely associated with a deficient release of sister chromatid cohesion (Ishii et al. 2010). However, it is unknown whether the impaired release of sister chromatid cohesion and centromere inactivity act together.

## Generation of haploids using targeted centromere manipulation

Ravi and Chan (2010) demonstrated in *A. thaliana* that manipulated centromeric histone protein CENH3 can mimic some of the outcomes of unstable inter- or intraspecific hybrids. Chromosomes of transgenic lines carrying modified CENH3 variants were shown to be eliminated during early embryogenesis, preferentially if the haploidy inducer was used as maternal parent. Besides haploidization, this approach can facilitate the transfer of a given nucleotype into a heterologous cytoplasm, e.g., conferring male sterility. Centromere-mediated haploidization has other potential applications: specifically, as a tool to accelerate the pyramiding of multiple mutants, as a forward mutagenesis screen, for down-sizing a polyploid to a lower ploidy level and for generating homozygotes for gametophyte-lethal mutations (Ravi et al. 2014). Doubled haploids can be exploited to rapidly generate mapping populations (Fulcher et al. 2015), chromosome substitution lines, parents for reverse breeding (Wijnker et al. 2014) and for the engineering of apomixis (Marimuthu et al. 2011). A combination of uniparental chromosome elimination and the MiMe-system (where meiosis is replaced by mitosis) has been proposed as a means to produce clonal seeds. In the study of Ravi and Chan (2010), a chimeric histone H3.3/CENH3 protein was constructed by fusing the N-terminal tail of a canonical *A. thaliana* H3.3 histone with a GFP reporter, and this construct was then accomplished by the addition of the C-terminal histone fold domain of CENH3. The resulting ‘GFP-tailswap’ protein complements the lethal phenotype of a *cenh3* null TILLING mutant. Between 25 and 45% of the progeny of the transgenic *CENH3* -*/*- haploidy inducer line is haploid when the inducer line is the maternal parent, whereas the induction rate drops to 5% when the haploidy inducer is used as a pollinator.

To elucidate whether, besides severe changes of CENH3, like the application of a CENH3-tailswap construct, a non-transgenic induced mutations in endogenous CENH3 could also used for haploidy induction, Karimi-Ashtiyani et al. (2015) screened a barley TILLING population for CENH3 mutations. The identified single point amino acid exchange in the CATD domain of βCENH3, leads to reduced centromere loading of this protein in barley, sugar beet and *A. thaliana*. Haploids were obtained after crossing wild-type Arabidopsis as pollinator with a *CENH3* L130F-complemented *cenh3* null mutant as seed parent. To better define CENH3 functional constraints, Kuppu et al. (2015) complemented the same *cenh3* null mutant of *A. thaliana* with a variety of mutant alleles, each inducing a single amino acid change in conserved positions of the histone fold domain. Many of these lines, while fertile upon self-pollination, produced uniparental haploids when crossed with wild-type plants. The high degree of evolutionary conservation of the identified CENH3 mutated region offers a promising opportunity for application in a wide range of crop species where haploid technology is still of limited availability.

Based on previous observations, a relationship between centromere size and haploid formation was proposed by Wang and Dawe (2018). In cases where a line with small or defective centromeres is crossed with a line featuring larger or normal centromeres, the smaller or defective centromeres are preferentially degraded, resulting in chromosome elimination from the parent possessing small chromosomes. In line with this model, a correlation between increased kinetochore protein and centromere strength was demonstrated for rearranged centromeres of mammals (Chmatal et al. 2014; Iwata-Otsubo et al. 2017). Artificial down-sizing of CENH3 resulting in haploids by targeted protein degeneration has been demonstrated in *Drosophila melanogaster* (Raychaudhuri et al. 2012). Cid protein (CENH3 in Drosophila) was depleted during spermatogenesis using the deGradFP system (Caussinus et al. 2011) based on the proteasome-mediated targeted degradation of the protein of interest. This principle may be applied in crops too, as targeted protein degradation by the 26S-proteasome was successfully demonstrated in *Nicotiana tabacum* (Baudisch et al. 2018). The identification of genes other than *CENH3* which could be used to trigger the formation of haploids is challenging, as a large genetic screen for haploidy inducers in *A. thaliana* has failed to identify any suitable mutants (Portemer et al. 2015).

## Application of the CENH3-based elimination process in crops

**Fig. 2:**
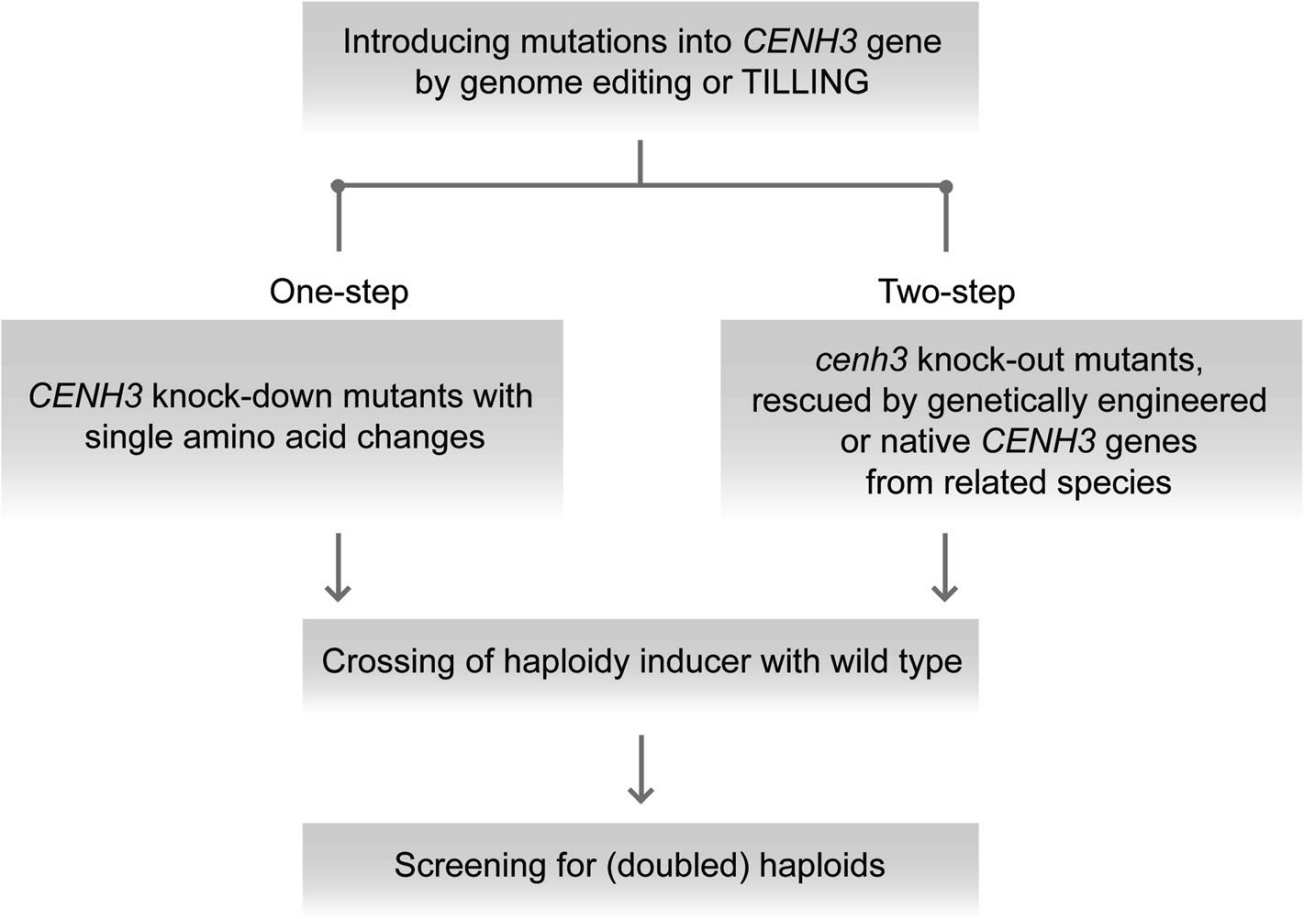
Schematic presentation of the two different strategies to create ‘haploidy inducer’ genotypes through manipulation of CENH3

Presently, CENH3-mediated haploid induction protocols leading to a reasonable number of haploids have been developed solely for the model plant *A. thaliana* (Karimi-Ashtiyani et al. 2015; Kuppu et al. 2015; Maheshwari et al. 2015; Ravi and Chan 2010). In crop species, an efficient CENH3-based haploid technology is not yet available, with the exception of maize where a rate of up to 3.6% haploids was achieved (Kelliher et al. 2016). Intense research is presently focused on this aim, and actually two different approaches are discussed for CENH3-based uniparental genome elimination (Fig. 2). The first approach is based on the two-step strategy presented by Ravi and Chan (2010), where lethal *A. thaliana* knockout *cenh3* mutants were successfully rescued by transformation with modified *CENH3* transgenes (Table 1). Later, it was demonstrated by the same group that functional complementation of *A. thaliana cenh3* null mutants and haploid induction is also possible with untagged natural CENH3 variants from progressively distant relatives such as different *Brassica* species (Maheshwari et al. 2015). This work also corroborated the earlier suggestion that evolutionarily close CENH3s are able to target centromeres in alien but not too far related species (Nagaki et al. 2010). The two-step strategy would most likely require the use of genetic modifications and would result in transgenic haploidy inducer lines. Although it is expected that the resultant haploid plants will be non-transgenic due to the loss of the chromosomes of the transgenic inducer plant, there is some concern especially in Europe, about the utilization of plant material that has undergone the process of genetic transformation within breeding programs.

**Table 1:** Survey of CENH3 modifications used to induce the formation of haploid plants

Haploidy inducer plants			Progeny		References
**Genetic background**	**CENH3 modification**	**Zygosity of modification**	**Haploids (f/m inducer)**	**Aneuploids (f/m inducer)**	
*Arabidopsis*					
T-DNA-interrupted cenh3	GFP-H3N-tail-CENH3 HFD on T-DNA	Homozygous	34%/4%	32%/11%	Ravi and Chan (2010)
T-DNA-interrupted cenh3	GFP-CENH3 on T-DNA	Homozygous	5%/0%	29%/4%	
T-DNA-interrupted cenh3	CENH3 P82S in HFD, on T-DNA	Homozygous	2.5%/–	–	Kuppu et al. (2015)
T-DNA-interrupted cenh3	CENH3 G83E in HFD, on T-DNA	Homozygous	10.5%/–	–	
T-DNA-interrupted cenh3	CENH3 A86V in HFD, on T-DNA	Homozygous	3.9%/–	–	
T-DNA-interrupted cenh3	CENH3 P102S in HFD, on T-DNA	Homozygous	0%/–	–	
T-DNA-interrupted cenh3	CENH3 A132T in HFD, on T-DNA	Homozygous	0.6%/–	–	
T-DNA-interrupted cenh3	CENH3 A136Tin HFD, on T-DNA	Homozygous	2.3%/–	–	
T-DNA-interrupted cenh3	CENH3 G173E in HFD, on T-DNA	Homozygous	0%/–	–	
WT	CENH3 A86V in HFD, EMS-induced	Homozygous	2.7%/–	–	
T-DNA-interrupted cenh3	CENH3 L130F in HFD, on T-DNA	Homozygous	4.8%/0%	8.4%/2.5%	Karimi-Ashtiyani et al. (2015)
*Barley*					
WT	L92F in HFD of ß-CENH3, EMS-induced	Homozygous	0%/0%	–	Karimi-Ashtiyani et al. (2015)
*Maize*					
CenH3 RNAi	GFP-H3N-tail-CENH3 HFD, on T-DNA	Homozygous	like WT/0.24 (max. 2.4)%	–	Kelliher et al. (2016)
CenH3 RNAi	GFP-CENH3, on T-DNA	Homozygous	like WT (max. 1.2)%/0%	–	
Transposon-interrupted cenH3	GFP-H3N-tail-CENH3 HFD, on T-DNA	Hemizygous	0.2%/0.86 (max. 3.6)%	1%/–	
Transposon-interrupted cenH3	GFP-CENH3, on T-DNA	Hemizygous	0.15%/0.32 (max 1.2)%	0%/–	
Transposon-interrupted cenH3	GFP-H3N-tail-CENH3 HFD, on T-DNA	Homozygous	0.13 (max. 1.2)%/0.13 (max.1.2)%	0%/–	
Transposon-interrupted cenH3	GFP-CENH3, on T-DNA	Homozygous	0.12 (max. 1.2)%/0.15 (max. 1.2)%	0%/–	
*Tomato*					
WT	K9E in N-tail, EMS-induced	Homozygous	0.2%/2.3%	0.2%/0%	WO 2017 200386/KEYGENE
*Rice*					
WT	CenH3 (K9E in N-tail), EMS-induced	Homozygous	1%/–	–	WO 2017 200386/KEYGENE
WT	CenH3 (P16S in N-tail), EMS-induced	Homozygous	0.3%/–	–	
WT	CenH3 (P26L in N-tail), EMS-induced	Homozygous	0.7%/–	–	
*Cucumber*					
WT	Premature STOP in HFD, EMS-induced	Hemizygous	1%/–	–	WO 2017 081011 A1/RIJK ZWAAN
*Melon*					
WT	CenH3 (D115V in HFD), EMS-induced	Homozygous	1.5%/–	–	WO 2017 081011 A1/RIJK ZWAAN

*f* female; *m* male

‘–’ not tested or data not provided

The alternative one-step approach is based on more or less targeted genetic modifications of the endogenous CENH3 gene. In a series of experiments, point mutations derived from TILLING approaches were selected, which were not lethal and yet responsible for reduced CENH3 loading to centromeres and/or the generation of so-called ‘weak centromeres’ in barley, sugar beet and *A. thaliana* (Karimi-Ashtiyani et al. 2015; Kuppu et al. 2015). As a viable alternative to the TILLING approach, *CENH3* genes are being modified via targeted mutagenesis using RNA-guided Cas9 endonucleases to generate partially functional *CENH3* alleles in the authors’ laboratories. Besides conventional genetic transformation using RNA-guided Cas endonuclease expression units, the recent establishment of using preassembled complexes of purified Cas9 protein and target gene-specific guide RNAs (Woo et al. 2015) opens up the opportunity to implement a particularly promising one-step modification of CENH3 without any transgene integration.

As a prerequisite for haploid formation using inducer lines carrying novel CENH3 variants, functional *CENH3* genes of the respective species must be cloned and analyzed for the identification of suitable modification sites within the hypervariable N-terminal region or the more conserved histone fold domain. In addition, if a two-step method is pursued, genetically engineered *CENH3* variants or alien *CENH3* genes have to be stably expressed to partially complement knockout mutations of the endogenous *CENH3*. So far, *CENH3* genes from several species have been cloned and characterized, e.g., rice (Hirsch et al. 2009), tobacco (Nagaki et al. 2009), soybean (Tek et al. 2010), *Brassica oleracea* (Wang et al. 2011), onion and garlic (Nagaki et al. 2012), pea (Neumann et al. 2012), common bean (Iwata et al. 2013), carrot (Dunemann et al. 2014), wheat (Yuan et al. 2015) and rye (Evtushenko et al. 2017). Nevertheless, to the best of our knowledge, protocols for haploid induction in other crop plants than maize, in which modifications within the CENH3-coding region were induced by mutagenesis, have not been published yet in the peer-reviewed literature. However, a patent from KEYGENE N.V. (Wageningen, Netherlands) describes haploidy inducers in tomato and rice (patent WO 2017/200386, (Op Den Camp et al. 2017)). In tomato, a non-synonymous mutation of A to G led to an exchange of lysine to glutamine at amino acid position 9 of the N-terminal region of CENH3. When this haploidy inducer line was used as pollinator of wild-type parents, as many as 4% of progeny proved to be doubled haploids. Homozygosity of these plants was determined via single nucleotide polymorphism analysis. In rice, three haploidy inducer lines, in which different single amino acids were substituted, resulted in up to 1% of haploid progeny. In addition, the Dutch breeding company Rijk Zwaan claims the production of haploid progeny in cucumber (*Cucumus sativu*s L.) and melon (*Cucumis melo* L.) (patent WO 2017/081011, (Van Dan et al. 2017)). In cucumber, haploidy inducer plants carrying a *CENH3* frame-shift mutation at heterozygous state were crossed with wild-type plants, which led to 1% of haploid or doubled haploid progeny. In melon, plants carrying homozygous mutations in the HFD of CENH3 were used for crossings with wild-type plants, which led to the formation of haploid or doubled haploid progeny at a frequency of 1.5%. A comprehensive survey of CENH3 variants used so far in plants to induce the formation of haploids is shown in Table 1.

The high number of reviews on CENH3-based haploidization (Britt and Kuppa 2016; Comai 2014; Copenhaver and Preuss 2010; Gilles et al. 2017b; Ishii et al. 2016; Ren et al. 2017; Watts et al. 2016) is diametric to the number of successful applications of this method in crop species. Thus, the expectations on the application of this approach in crops are high while the establishment of viable CENH3-based haploidization methods is challenging.

In conclusion, novel promising tools for the generation of haploids are becoming available. With the recent identification of a patatin-like phospholipase mutation, the gene behind the Stock6-derived maternal haploid induction systems of maize, a similar tool could become possible at least in other monocotyledons. The improvement and implementation of CENH3-based haploidization approaches in crop breeding remains a mission to be fulfilled.

### Author contribution statement

All authors contributed towards this manuscript.
